# Screening of gene markers related to the prognosis of metastatic skin cutaneous melanoma based on Logit regression and survival analysis

**DOI:** 10.1186/s12920-021-00923-0

**Published:** 2021-04-06

**Authors:** Guoliang Jia, Zheyu Song, Zhonghang Xu, Youmao Tao, Yuanyu Wu, Xiaoyu Wan

**Affiliations:** 1 Department of Orthopedics, The Second Clinical Hospital of Jilin University, NO.218, Ziqiang Street, Nanguan District, Changchun, 130000 Jilin China; 2 Department of Gastrointestinal and Colorectal Surgery, The Third Hospital of Jilin University, No.126, Xiantai Street, Changchun, 130033 Jilin China; 3 Department of Brest Surgery, The Second Clinical Hospital of Jilin University, NO.218, Ziqiang Street, Nanguan District, Changchun, 130000 Jilin China

**Keywords:** Cutaneous melanoma, Metastasis, Prognosis, Bioinformatics, Differentially expressed genes

## Abstract

**Background:**

Bioinformatics was used to analyze the skin cutaneous melanoma (SKCM) gene expression profile to provide a theoretical basis for further studying the mechanism underlying metastatic SKCM and the clinical prognosis.

**Methods:**

We downloaded the gene expression profiles of 358 metastatic and 102 primary (nonmetastatic) CM samples from The Cancer Genome Atlas (TCGA) database as a training dataset and the GSE65904 dataset from the National Center for Biotechnology Information database as a validation dataset. Differentially expressed genes (DEGs) were screened using the limma package of R3.4.1, and prognosis-related feature DEGs were screened using Logit regression (LR) and survival analyses. We also used the STRING online database, Cytoscape software, and Database for Annotation, Visualization and Integrated Discovery software for protein–protein interaction network, Gene Ontology, and Kyoto Encyclopedia of Genes and Genomes (KEGG) pathway analyses based on the screened DEGs.

**Results:**

Of the 876 DEGs selected, 11 (ZNF750, NLRP6, TGM3, KRTDAP, CAMSAP3, KRT6C, CALML5, SPRR2E, CD3G, RTP5, and FAM83C) were screened using LR analysis. The survival prognosis of nonmetastatic group was better compared to the metastatic group between the TCGA training and validation datasets. The 11 DEGs were involved in 9 KEGG signaling pathways, and of these 11 DEGs, CALML5 was a feature DEG involved in the melanogenesis pathway, 12 targets of which were collected.

**Conclusion:**

The feature DEGs screened, such as CALML5, are related to the prognosis of metastatic CM according to LR. Our results provide new ideas for exploring the molecular mechanism underlying CM metastasis and finding new diagnostic prognostic markers.

## Background

Skin cutaneous melanoma (SKCM) is a common skin malignancy with poor prognosis due to aggressiveness and metastasis [1]. In recent years, the incidence of SKCM has significantly increased, and the survival rate of patients remains poor; the 5-year survival rate of metastatic SKCM patients is less than 5% [2]. As metastasis is an important cause of poor prognosis of SKCM, it is necessary to identify its underlying molecular mechanisms and determin its molecular biomarkers.

Previous studies have focused on molecular markers related to SKCM metastasis and prognosis. For example, S100 has been clearly identified as a diagnostic marker of SKCM, and in terms of SKCM prognosis, the most studied diagnostic marker is S100B. Other studies have reported that high S100B expression indicates tumor recurrence and metastasis [3, 4]. Da Forno et al. [5] showed that high expression of Wnt-5a indicates an increase in SKCM aggressiveness, distant metastases, and poor prognosis. Melanoma inhibitory protein is also considered a diagnostic marker of SKCM metastasis and poor prognosis [6]. Ci et al. [7] showed that CDCA8 overexpression promotes the malignant progression of SKCM and leads to poor prognosis. Yang et al. [8] found that *STK26*, *KCNT2*, and *CASP12* expression is correlated with the prognosis of SKCM using weighted gene co-expression network analysis. However, the mechanism underlying metastasis of a nevus into SKCM is still unclear, and further research is urgently needed.

Logit regression (LR) is popular among medical practitioners because of its interpretability and ease of application without the need for a computer [9]. Zejnullahu et al. [10] examined the prevalence and risk factors of postpartum depression using LR. Sufriyana et al. [11] used LR to explore the predictive performances for pregnancy care to inform clinicians’ decision making. Sokou et al.[12] developed and validated a prediction model for clinical variables. These reports suggested that LR model for predicting the risk assessment is regularly used in medicine to guide management decisions.

In this study, we downloaded gene expression detection data of 460 (358 metastatic and 102 primary [nonmetastatic]) SKCM patients from The Cancer Genome Atlas (TCGA) database. The limma package of R3.4.1 was used to screen for differentially expressed genes (DEGs), the survival package of R3.4.1 was used for single- and multifactor Cox regression analysis, and feature DEGs were selected using LR. Next, we constructed a protein–protein interaction (PPI) network, followed by Gene Ontology (GO) and Kyoto Encyclopedia of Genes and Genomes (KEGG) pathway enrichment analyses.

## Methods

### Gene expression profile data and clinical information

We downloaded 472 SKCM patients’ gene expression profile data using Illumina HiSeq 2000 from the TCGA database (https://gdc-portal.nci.nih.gov/). According to the clinical information, 460 tumor samples had information about metastasis, of which 358 metastatic and 102 nonmetastatic SKCM tumor samples were used as a training dataset. Table 1 showed all the clinical information present in the dataset; there was significant association in pathologic N status, tumor recurrence, and death between metastatic SKCM and nonmetastatic SKCM. In addition, we downloaded another data set of SKCM patient gene expression profiles (GSE65904) from the National Center for Biotechnology Information database (NCBI) (https://www.ncbi.nlm.nih.gov/), which contained 214 SKCM tumor samples. The detection platform used was the Illumina HumanHT-12 V4.0 expression beadchip. According to the clinical information, 150 tumor samples had information about metastasis, of which 83 metastatic and 67 nonmetastatic SKCM tumor samples were used as a validation dataset.

**Table 1:** Statistical comparison of clinical information of two types of CM samples in TCGA training data set

Clinical characteristics	With metastatic (N = 358)	Without metastatic (N = 102)	*P* value
Age (years, mean ± sd)	56.26 ± 15.73	64.53 ± 13.91	7.10E−07^a^
Gender (Male/Female)	226/132	60/42	0.488^b^
Pathologic_M (M0/M1/–)	313/21/24	97/3/2	0.317^b^
Pathologic_N (N0/N1/N2/N3/–)	172/65/39/44/38	57/8/10/11/16	0.0785^b^
Pathologic_T (T0/T1/T2/T3/T4/–)	23/40/72/80/67/76	0/1/5/10/84/2	2.2E−16^b^
Pathologic_stage (I/II/III/IV/–)	81/74/142/20/41	2/65/27/3/5	1.133E−15^b^
Radiotherapy (Yes/No/–)	47/310/1	2/100/0	4.395E−04^b^
Tumor recurrence (Yes/No/–)	69/135/154	20/72/10	0.0402^b^
Dead (Death/Alive)	193/165	29/73	0.0715^b^
Overall survival time (months, mean ± sd)	74.10 ± 67.91	47.15 ± 10.04	5.279E−22^a^

^a^T test between groups

^b^Fisher exact test

### Data preprocessing and DEG screening

Tumor samples in the training dataset were divided into metastatic and nonmetastatic groups according to the clinical information. DEGs in both groups were analyzed using the limma package (https://bioconductor.org/packages/release/bioc/html/limma.html) version 3.34.7 of R3.4.1. FDR < 0.05 and |log_2_FC|> 1 were used as the cut-off threshold.

Next, we performed two-way hierarchical clustering based on the centered Pearson correlation algorithm based on the DEG expression in the training dataset using pheatmap version 1.0.8 (https://cran.r-project.org/web/packages/pheatmap/index.html) of R3.4.1, and then performed the following analysis:

1. To determine whether the clinical information about the tumor samples in different clusters was significantly different, we ran the chisq.test function (http://www.bioconductor.org/help/search/index.html?q=chisq.test/) of R3.4.1 on the clinical information.
2. According to the survival prognosis information about tumor samples in different clusters, we used the Kaplan–Meier (KM) curve in the survival package version 2.41–1 (http://bioconductor.org/packages/survivalr/) of R3.4.1 to calculate the correlation between clusters and survival prognosis.

### Screening of independent prognostic DEGs

On the basis of the clinical prognosis information about the SKCM tumor samples included in this analysis, we univariate Cox regression analysis in the survival package version 2.41–1 of R3.4.1 [13] to screen DEGs with significant differences in prognosis. We further used multivariate Cox regression analysis to screen independent prognosis-related DEGs, and log-rank *P* value < 0.05 was selected as the threshold for significant correlation.

### Screening of feature DEGs

On the basis of previously screened DEGs independently associated with survival prognosis, we used the glm function of R3.4.1 to perform LR [14, 15] to screen out feature DEGs and classify metastatic and nonmetastatic SKCM tumor samples. All genes with *P* < 0.05 were considered feature DEGs. Next, on the basis of the expression of the feature DEGs, we calculated the discriminant accuracy and classified all tumor samples in the training and validation datasets into metastatic and nonmetastatic groups. The KM survival curve in the survival package version 2.41-1 of R3.4.1 was used to perform correlation analysis of the actual survival prognosis of the metastatic and nonmetastatic SKCM tumor samples obtained from the LR classification.

### Cluster analysis of feature DEGs

For the training and validation datasets, we used pheatmap version 1.0.8 [16] of R3.4.1 to perform two-way hierarchical clustering based on the centered Pearson correlation algorithm based on feature DEG expression. On the basis of the survival prognosis information about samples in different clusters, we used the KM survival curve in the survival package version 2.41-1 (http://bioconductor.org/packages/survivalr/) of R3.4.1 to determine the correlation between clusters and survival prognosis in the training and validation datasets. The expression levels of feature DEGs in different clusters were then demonstrated.

### PPI network construction function analysis

We constructed a PPI network to evaluate the interactions between proteins encoded by the DEGs. The PPI network was based on the STRING database version 10.0 (http://string-db.org/) [17] and was visualized using Cytoscape software version 3.6.1 (http://www.cytoscape.org/) [18]. Next, we used the Database for Annotation, Visualization and Integrated Discovery (DAVID) version 6.8 (https://david.ncifcrf.gov/) to conduct GO function annotation, including Biology Process, and KEGG signaling pathway enrichment analysis [19, 20]. Finally, significantly relevant GO functions were screened using Fisher’s exact test with *P* < 0.05.

## Results

### DEG screening

**Fig. 1:**
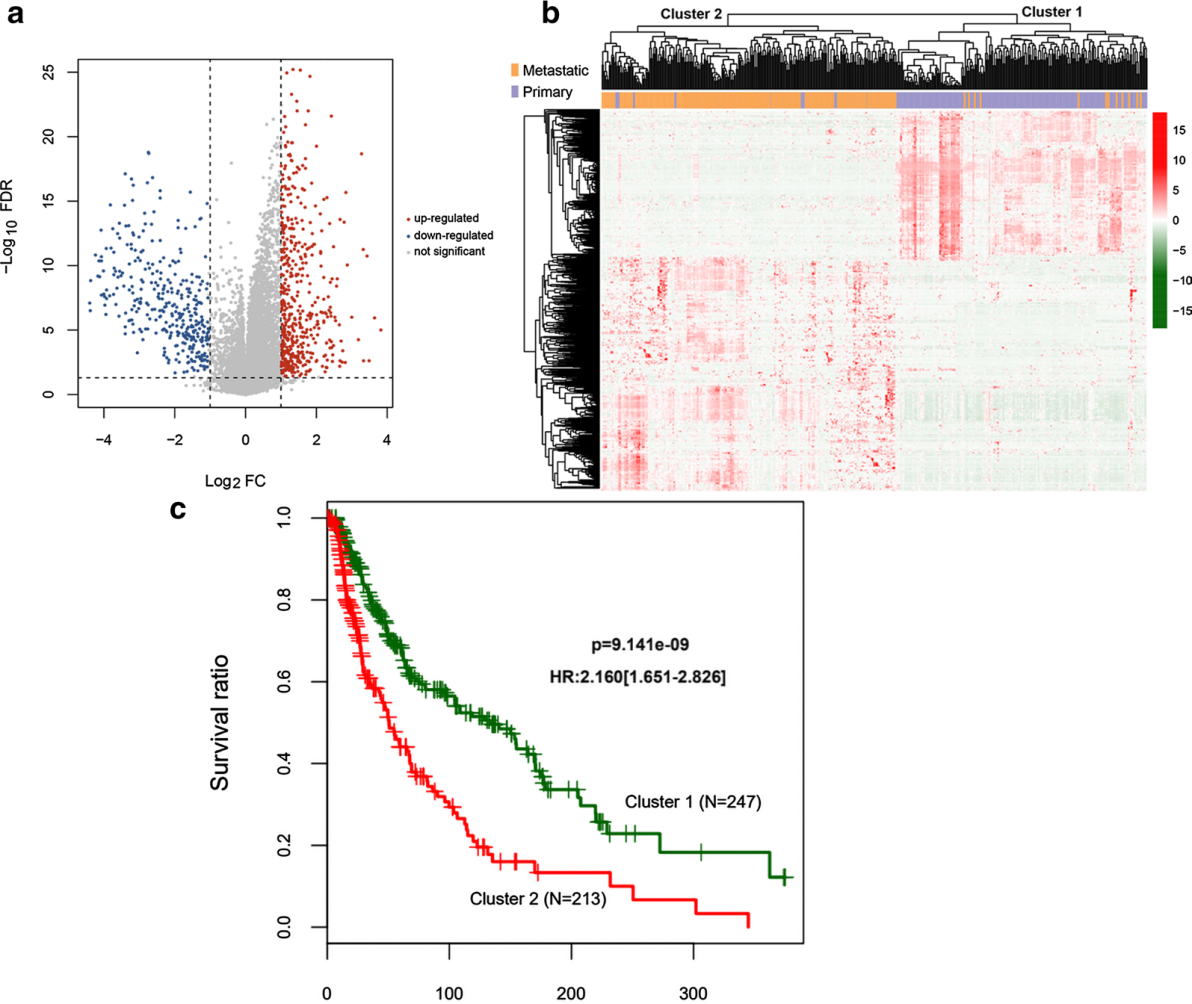
DEGs screening. **a** Volcano map of DEGs. The blue and red dots indicate DEGs that are significantly downregulated and upregulated, respectively. The black horizontal line indicates FDR < 0.05, and the two black vertical lines indicate |log2FC|> 1. **b** Two-way hierarchical clustering heat map based on DEG expression. In the sample bar, orange and purple represent metastatic and nonmetastatic SKCM tumor samples, respectively. **c** KM curves of sample prognosis information about cluster 1 and cluster 2, which is obtained by clustering based on DEG expression in the TCGA training dataset. The green and red curves represent cluster 1 and cluster 2 tumor sample groups, respectively. DEGs, differentially expressed genes; SKCM, skin cutaneous melanoma; KM, Kaplan–Meier; TCGA, The Cancer Genome Atlas

On the basis of the clinical information, SKCM tumor samples in the TCGA training dataset were divided into metastatic (*n* = 358) and nonmetastatic (*n* = 102) groups. We screened 876 DEGs, including 353 significantly downregulated and 523 significantly upregulated DEGs. Figure 1a showed the test volcano map. The two-dimensional hierarchical clustering heat map (Fig. 1b) showed that samples with similar gene expression patterns were hierarchically clustered into the same group, indicating that the screened DEGs can well distinguish tumor samples with different prognoses. The two clusters of the clustering chart contained 247 and 213 tumor samples, respectively. Comparing the clinical information of the tumor samples in the two clusters showed that the samples were significantly different in age, pathologic T status, pathologic stage, radiotherapy, tumor metastasis, and survival time (Table 2). In addition, comparing the prognostic levels of the tumor samples showed that the survival ratio of cluster 1 was significantly better than cluster 2 (*P* = 9.141e−09) (Fig. 1c).

**Table 2:** Statistical comparison of clinical information among samples in clusters based on DEGs expression levels

Clinical characteristics	Cluster 1 (N = 247)	Cluster 2 (N = 213)	*P* value
Age (years, mean ± sd)	54.96 ± 15.41	61.74 ± 15.29	3.07E−06^a^
Gender (Male/Female)	152/95	134/79	0.773^b^
Pathologic_M (M0/M1/–)	219/14/14	191/10/12	0.679^b^
Pathologic_N (N0/N1/N2/N3/–)	118/42/28/33/26	111/31/21/22/28	0.599^b^
Pathologic_T (T0/T1/T2/T3/T4/–)	20/33/51/54/44/45	3/8/26/36/107/33	2.893E−13^b^
Pathologic_stage (I/II/III/IV/–)	66/43/97/14/27	17/96/72/9/19	6.568E−12^b^
Radiotherapy (Yes/No/–)	35/212/0	14/198/1	0.00975^b^
Tumor recurrence (Yes/No/–)	45/107/95	44/100/69	0.899^b^
Tumor metastatic (Yes/No)	235/12	123/90	2.200E−16^b^
Dead (Death/Alive)	111/136	111/102	0.149^b^
Overall survival time (months, mean ± sd)	78.21 ± 71.12	42.07 ± 49.53	4.269E−10^a^

^a^T test between groups

^b^Fisher exact test

### Screening of independent prognostic DEGs

On the basis of the clinical prognosis information about the 460 SKCM tumor samples, we analyzed the 876 screened DEGs by univariate single-factor Cox regression analysis using the survival package version 2.41-1 of R3.4.1 and screened 435 DEGs significantly associated with prognosis. These 435 DEGs were further analyzed using multivariate Cox regression analysis, and 61 independent prognostic DEGs were screened.

### Screening of feature DEGs by LR

**Fig. 2:**
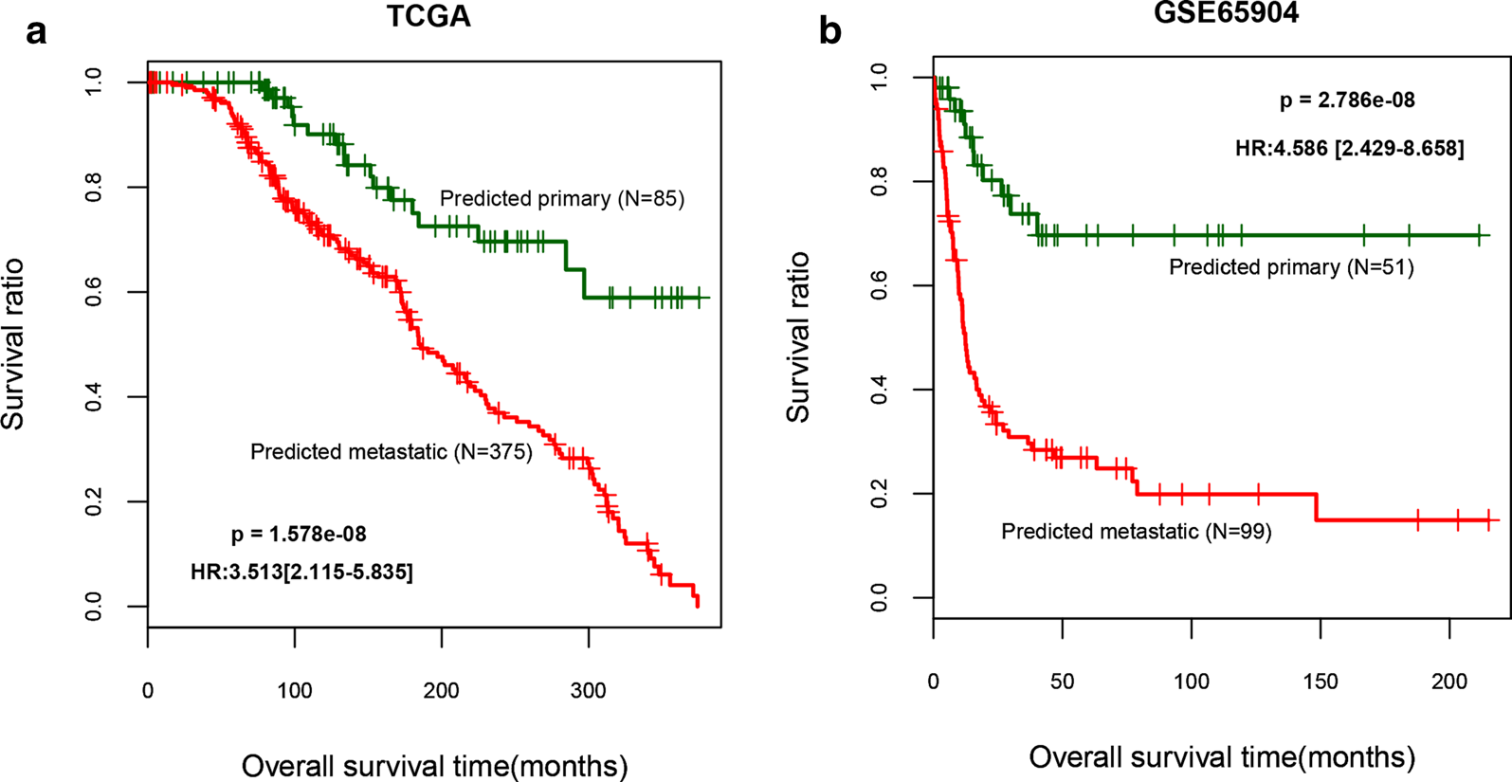
KM curves of survival prognosis based on LR classification results for the TCGA training dataset (**a**) and the GSE65904 validation dataset (**b**). The green and red curves represent the predicted SKCM tumor samples of nonmetastatic and metastatic types, respectively. LR, Logit regression; SKCM, skin cutaneous melanoma; KM, Kaplan–Meier; TCGA, The Cancer Genome Atlas

The 61 independent prognostic DEGs were further screened using LR to obtain 11 feature DEGs: *ZNF750*, *NLRP6*, transglutaminase 3 (*TGM3*), *KRTDAP*, *CAMSAP3*, *KRT6C*, *CALML5*, *SPRR2E*, *CD3G*, *RTP5*, and *FAM83C* (Table 3). LR was also used to classify the tumor samples in the training and validation datasets on the basis of the expression levels of these 11 feature DEGs (Table 4). Metastatic SKCM accounted for 98% of the tumor samples in the training dataset and 87% of the tumor samples in the validation dataset. In addition, KM curve analysis was performed on the basis of LR classification prediction results (Fig. 2a, b). In the TCGA training and GSE65904 validation datasets, the predicted survival prognosis of the nonmetastatic group was better than the metastatic group (*P* = 1.578e−08 and 2.786e−08, respectively).

**Table 3:** List of important prognostic feature DEGs screened by Logit model

Gene	B	SE	Df	*P* value
ZNF750	− 7.07556	3.11741	1	0.02323
NLRP6	− 3.81707	1.92378	1	0.04724
TGM3	− 3.63216	1.08106	1	0.00078
KRTDAP	− 1.36009	0.65739	1	0.03855
CAMSAP3	− 1.18449	0.52553	1	0.0242
KRT6C	− 0.96555	0.44870	1	0.03141
CALML5	1.47087	0.57774	1	0.0109
SPRR2E	1.59208	0.53097	1	0.00271
CD3G	4.12233	1.77857	1	0.02046
RTP5	7.35929	2.91801	1	0.01167
FAM83C	9.35699	3.95983	1	0.01813

B, regression coefficient; SE, standard error; Df, degree of freedom

**Table 4:** Fuzzy matrix of LR classification result

Dataset name	Predict			
	Class	Metastatic	Primary	Percent
TCGA dataset	Observed			
	Metastatic	350	8	97.77
	Primary	25	77	75.49
Overall percent				92.83
GSE65904 microarray dataset	Observed			
	Metastatic	72	11	86.75
	Primary	27	40	59.70
Overall percent				74.67

### Cluster analysis of feature DEGs

**Fig. 3:**
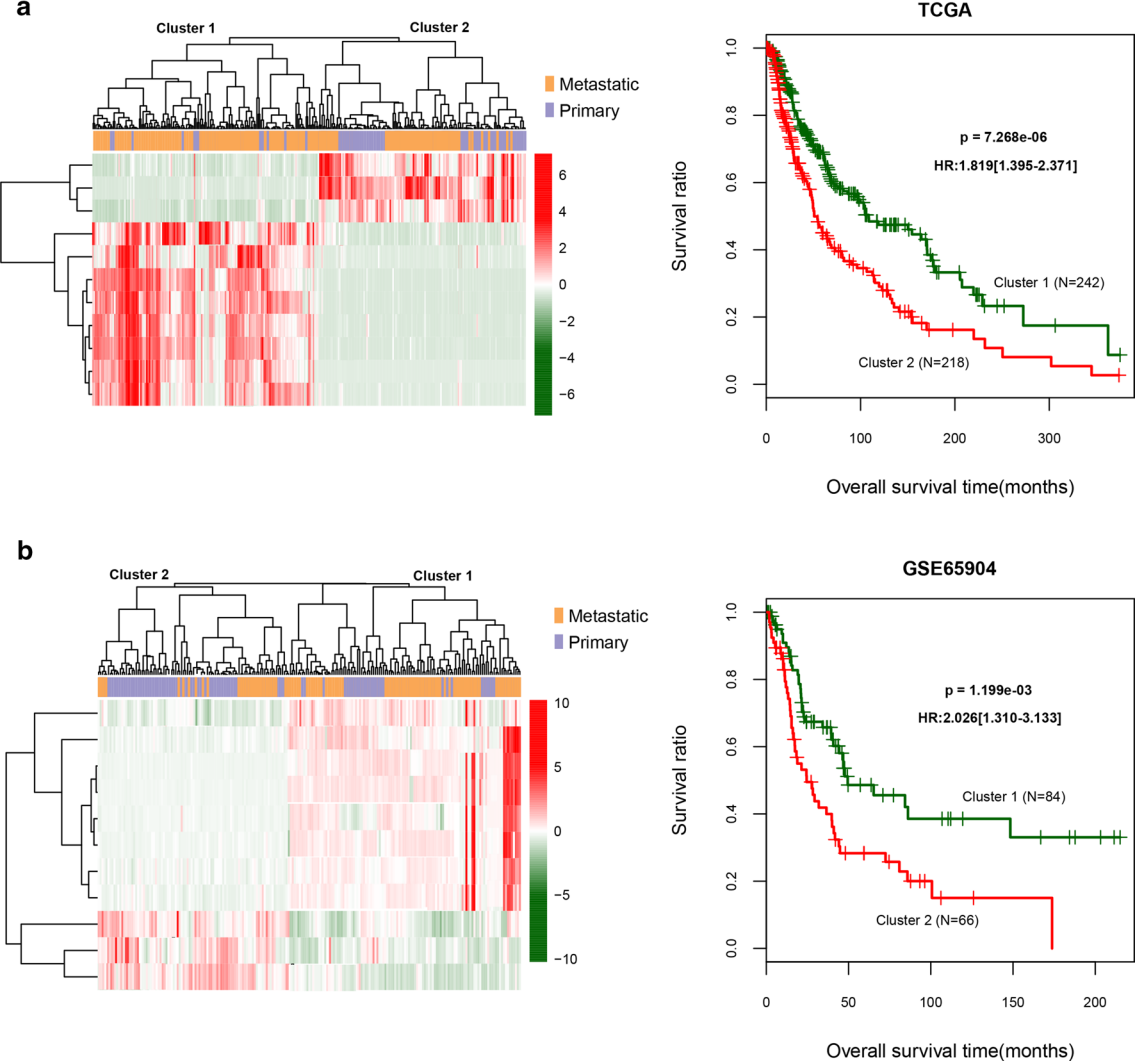
TCGA training dataset (**a**) and GSE65904 validation dataset (**b**). Left: Two-way hierarchical clustering chart based on the expression of 11 feature DEGs. Right: KM curve of sample prognosis information about cluster 1 and cluster 2 based on left clustering. The green and red curves represent the cluster 1 and cluster 2 tumor sample groups, respectively. DEGs, differentially expressed genes; KM, Kaplan–Meier; TCGA, The Cancer Genome Atlas

**Fig. 4:**
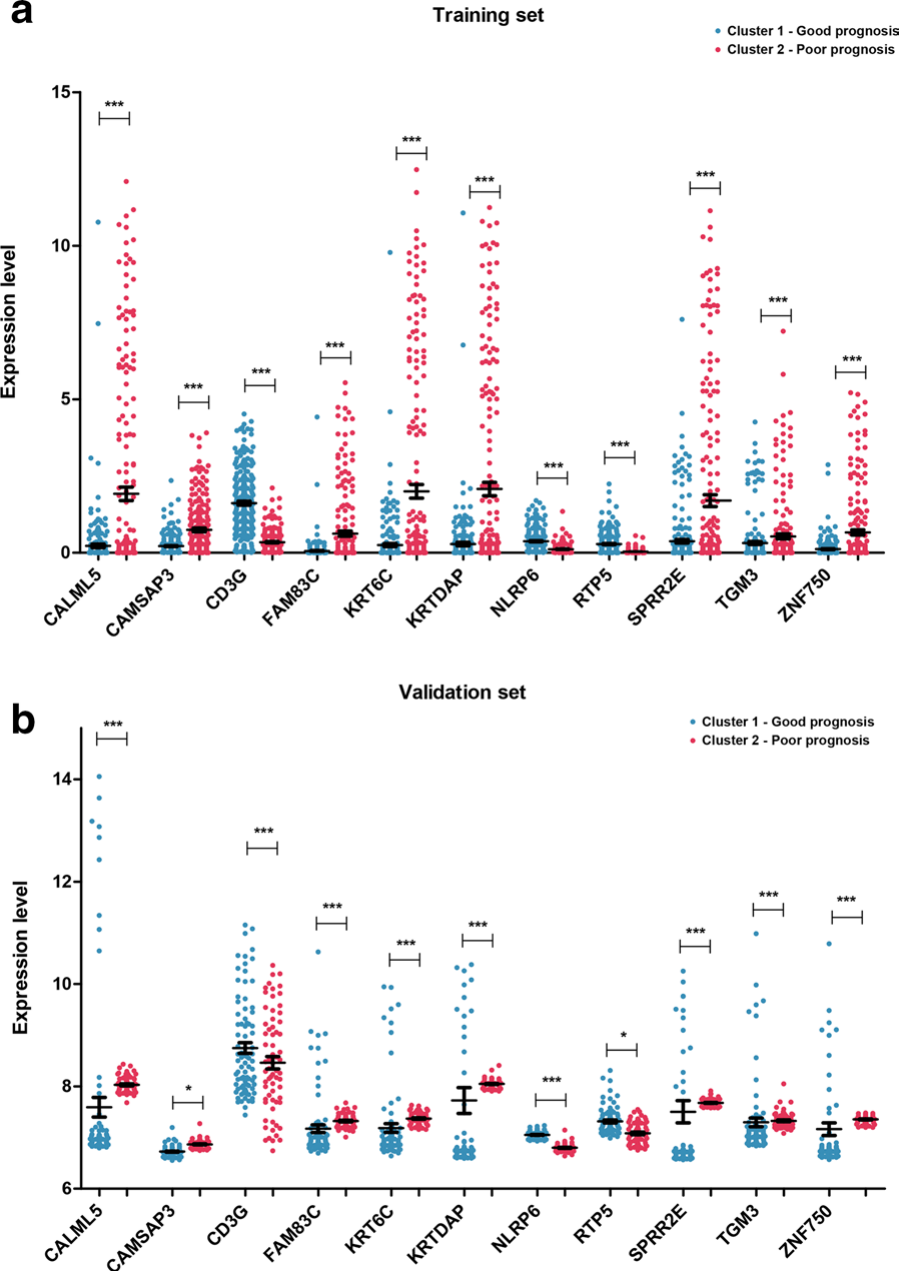
Expression of 11 feature DEGs in different clusters in the TCGA training dataset (**a**) and the GSE65904 validation dataset (**b**). The blue and red dots represent the tumor samples in clusters 1 and 2, respectively. DEGs, differentially expressed genes; TCGA, The Cancer Genome Atlas

In the TCGA training and GSE65904 validation datasets, we performed two-way hierarchical clustering based on the centered Pearson correlation algorithm based on the expression of the 11 feature DEGs (Fig. 3a). We also performed KM curve analysis on the tumor samples in clusters 1 and 2 on the basis of the expression of the 11 DEGs. In TCGA the training and GSE65904 validation datasets, the survival prognosis in cluster 1 was better compared to cluster 2 (*P* = 7.268e−06 and 1.199e−03, respectively) (Fig. 3b). The expression levels of the 11 feature DEGs in the two clusters of the TCGA training and GSE65904 validation datasets are shown in Fig. 4. Tumor samples with similar gene expression patterns were hierarchically clustered into the same group, indicating that the selected DEGs can well distinguish tumor samples with different prognoses. We identified eight genes (*CALML5*, *CAMSAP3*, *FAM83C*, *KRTDAP*, *SPRR2E*, *TGM3*, and *ZNF750*) with high expression associated with poor prognosis and three genes (*CD3G*, *NLRP6*, and *RTP5*) with low expression associated with poor prognosis (Fig. 4a). In the GSE65904 validation dataset, we obtained similar results: three genes (*CD3G*, *NLRP6*, and *RTP5*) with low expression were associated with poor prognosis (Fig. 4b).

### PPI analysis

**Fig. 5:**
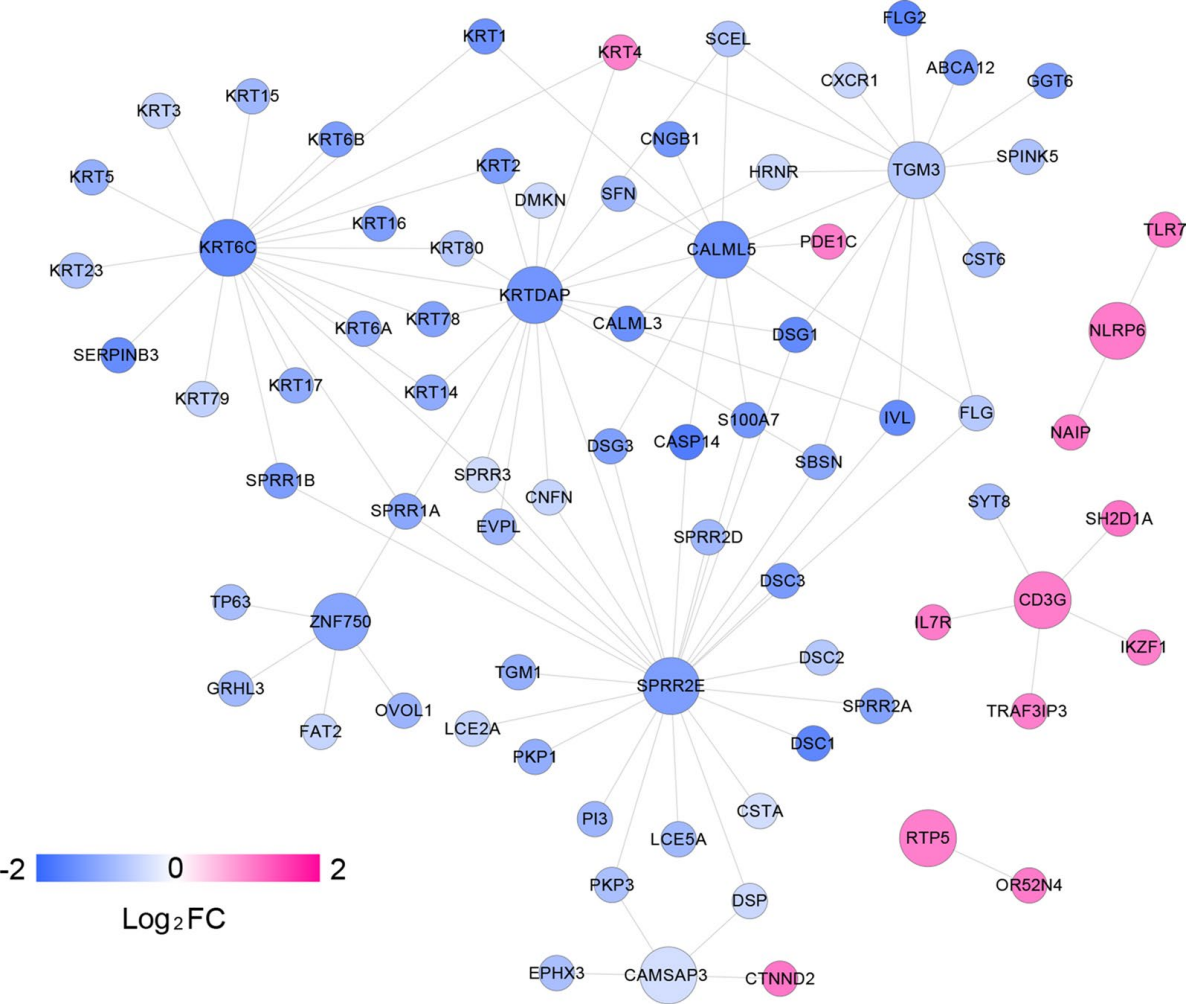
PPI network diagrams related to the feature DEGs. The change in color from blue to red indicates the change in expression from low to high in the comparison of metastatic vs. nonmetastatic SKCM, and the larger nodes indicate feature DEGs. PPI, protein–protein interaction; DEGs, differentially expressed genes; SKCM, skin cutaneous melanoma

We constructed a PPI network based on the 876 screened DEGs using the STRING online database. After screening the 11 feature DEGs using LR, we only focused on their PPI network, which contained 107 pairs of interaction connections related to the 11 feature DEGs. Cytoscape software was used to construct the PPI network (Fig. 5) containing 78 nodes (65 downregulated and 13 upregulated) and 107 edges. We collected 12 targets of *CALML5*, including *TGM3*, *PDE1C*, *CASP14*, and *CASP14*, and 26 targets of *SPRR2E*, including *SPRR2A*, *IVL*, and *LCE2A*.

### GO and KEGG signaling pathway enrichment analysis

We obtained 21 significantly related GO biological processes and 9 KEGG signaling pathways using the online tool DAVID (Table 5). For GO functions, the DEGs were primarily enriched in “epidermis development,” “keratinization,” “keratinocyte differentiation,” and “establishment of skin barrier,” which involved many genes, such as *CALML5*, *SPRR2A*, and *HRNR*. For KEGG signaling pathways, the DEGs were primarily enriched in “calcium signal pathway,” “GnRH signal pathway,” and “melanin production pathway.” Of the 9 KEGG signaling pathways, in the “hsa04916: Melanogenesis” pathway, *CALML3* and *CALML5* deserved attention, of which *CALML5* was one of important feature DEGs.

**Table 5:** GO biological processes and KEGG signaling pathways in which genes are significantly related in the interaction network

Category	Term	Count	Gene	*P* value
Biology Process	GO:0,031,424 ~ keratinization	20	TGM3, HRNR	3.18E−35
	GO:0,008,544 ~ epidermis development	22	CALML5, ZNF750	9.46E−34
	GO:0,030,216 ~ keratinocyte differentiation	19	TGM3, TP63	1.04E−28
	GO:0,018,149 ~ peptide cross-linking	14	LCE5A, EVPL	8.98E−22
	GO:0,061,436 ~ establishment of skin barrier	9	HRNR, FLG	2.25E−16
	GO:0,045,104 ~ intermediate filament cytoskeleton organization	4	KRT6C, KRT3	2.92E−06
	GO:0,043,163 ~ cell envelope organization	3	HRNR, TGM3	4.98E−06
	GO:0,016,337 ~ single organismal cell–cell adhesion	6	PKP1, DSG1	5.90E−06
	GO:0,042,060 ~ wound healing	5	NLRP6, KRT6A	3.26E−05
	GO:0,045,109 ~ intermediate filament organization	3	KRT17, KRT2	1.92E−04
	GO:0,030,855 ~ epithelial cell differentiation	4	KRT14, KRT3	2.99E−04
	GO:0,007,010 ~ cytoskeleton organization	5	KRT6B, KRT5	4.34E−04
	GO:0,031,069 ~ hair follicle morphogenesis	3	KRT17, TGM3	5.47E−04
	GO:0,010,482 ~ regulation of epidermal cell division	2	TP63, SFN	1.23E−03
	GO:0,045,110 ~ intermediate filament bundle assembly	2	PKP1, KRT14	1.23E−03
	GO:0,051,546 ~ keratinocyte migration	2	KRT16, KRT2	2.04E−03
	GO:0,045,087 ~ innate immune response	6	NLRP6,SH2D1A	3.18E−03
	GO:0,010,838 ~ positive regulation of keratinocyte proliferation	2	TGM1, TP63	3.64E−03
	GO:0,003,334 ~ keratinocyte development	2	KRT2, SFN	4.04E−03
	GO:0,031,581 ~ hemidesmosome assembly	2	KRT, KRT14	4.82E−03
	GO:0,043,616 ~ keratinocyte proliferation	2	TP63, KRT2	4.82E−03
KEGG Pathway	hsa04020:Calcium signaling pathway	3	CALML3, CALML5	9.31E−03
	hsa04070:Phosphatidylinositol signaling system	2	CALML3, CALML5	1.98E−02
	hsa04640:Hematopoietic cell lineage	2	CD3G, IL7R	2.26E−02
	hsa04912:GnRH signaling pathway	2	CALML3, CALML5	2.53E−02
	hsa04916:Melanogenesis	2	CALML3, CALML5	2.56E−02
	hsa04114:Oocyte meiosis	2	CALML3, CALML5	2.80E−02
	hsa04270:Vascular smooth muscle contraction	2	CALML3, CALML5	2.84E−02
	hsa04722:Neurotrophin signaling pathway	2	CALML3, CALML5	3.10E−02
	hsa04910:Insulin signaling pathway	2	CALML3, CALML5	3.32E−02

## Discussion

SKCM is the most malignant skin tumor type derived from melanocytes, with high mortality, high metastasis, and difficulty in treating. As SKCM grows, cancer cells penetrate the skin and mucous membranes, eventually reach blood vessels or lymphatic channels, and quickly spread throughout the body and major organs [21]. Metastasis is an important cause of poor prognosis in SKCM patients [22]. The 5-year survival rate of metastatic SKCM patients is only 4.6%. Therefore, there is an urgent need to identify potential prognosis-related genes for SKCM and provide more powerful gene markers for the diagnosis of metastatic prognosis in SKCM patients.

In this study, we performed a comprehensive bioinformatics analysis based on the gene expression profile datasets (TCGA and GSE65904 databases) of SKCM patients. By classifying and comparing the gene expression data in the training dataset, we found 876 DEGs, of which 353 were downregulated and 523 were upregulated. With univariate and multivariate Cox regression analysis in the survival package of R3.4.1, we screened 61 independent prognostic DEGs, and then using LR and cluster analysis, we successfully screened 11 feature DEGs: *ZNF750*, *NLRP6*, *TGM3*, *KRTDAP*, *CAMSAP3*, *KRT6C*, *CALML5*, *SPRR2E*, *CD3G*, *RTP5*, and *FAM83C*. From among these 11 feature DEGs, we screened feature DEGs that significantly increased or decreased between cluster 1 and cluster 2 in the training dataset with *P* < 0.001. To understand the biological functions of the screened DEGs, we used the STRING database to search for interactions between the products of screened DEGs and constructed a PPI network. Then, we used the DAVID online tool perform GO function and KEGG signaling pathway enrichment analysis of genes on the PPI network. Of the screened 9 KEGG signaling pathways, one is worth pointing out: “hsa04916: Melanogenesis.” There are two genes involved in this pathway, *CALML3* and *CALML5*, of which *CALML5* is one of the important feature DEGs.

*CALML5* encodes the CALML5 protein with 146 amino acids. Unlike the generally expressed calmodulin, CALML5 expression is limited to the epidermis and other layered epithelial tissues, and it is highly expressed in the differentiated epidermis [23]. In this study, we found that *CALML5* is involved in epidermal development, the calcium signaling pathway, and vascular smooth muscle contraction. Previous studies have reported that *CALML5* is involved in terminal differentiation of keratinocytes and encodes a calcium-binding protein expressed in the epidermis [24]. In addition, CALML5, as a calmodulin-like protein, participates in not only epidermal differentiation but also intracellular signal transduction. Therefore, *CALML5* plays an important role in cell proliferation, differentiation, apoptosis, and migration [25]. Kurozumi et al. showed that *CALML5* is a key gene for lymphatic vascular infiltration in early breast cancer and has potential prognostic value [26]. In addition, *CALML5* is involved in the “hsa04916: Melanogenesis” pathway. Mac et al. [27] showed that calmodulin-like protein can the SKCM cell growth. In addition, Ke et al. [28] found that *CALML5* may be considered a novel biomarker for lung adenocarcinoma diagnosis, which is useful for predicting the risk of lung adenocarcinoma. Misawa et al. [29] found that *CALML5* has a high predictive ability as an emerging biomarker for a validation set, capable of discriminating between the plasma of patients and healthy individuals. However, a few studies have reported an association between *CALML5* and SKCM, although the mechanism is unclear. Our results will provide a novel view for the prognosis of SKCM.

In this study, we collected 12 targets of *CALML5*, including *TGM3*, *PDE1C*, and *CASP14*. Smirnov et al. [30] showed that *TGM3* is absent in melanocytes as well as SKCM samples and that the expression pattern of *TGM3* renders it a potential specific marker for basal cell carcinoma diagnosis. Hu et al. [31] reported that *TGM3* controls multiple oncogenic pathways in hepatocellular carcinoma (HCC), contributing to increased cell proliferation and epithelial–mesenchymal transition (EMT). *TGM3* also potentially enhances HCC metastasis. *TGM3* may serve as a novel therapeutic target in HCC. Wu et al. [32] found that *PDE1C* is associated with SKCM development. Shimizu et al. found that *PDE1C* messenger RNA is expressed and may play an important role in human malignant SKCM melanoma-associated antigen cells. Chen et al. [33] reported that *CASP14* might be a potential biomarker for gastric cancer diagnosis and an independent prognostic factor of gastric cancer. These results indicate that *TGM3* may be an important target gene for SKCM treatment. Thus, *CALML5* may play an important role in SKCM metastasis and promote SKCM occurrence and development, so *CALML5* may become a potential target for the treatment of SKCM metastasis in the future.

Another calmodulin-like protein, *CALML3*, is a tumor suppressor gene, which significantly inhibits liver cancer growth and lung metastasis. *CALML3* is a gene with “early warning” value for liver cancer and lung metastasis, and it is expected to become a new marker for early diagnosis of lung metastasis of liver cancer and a new target for inhibiting liver cancer growth and lung metastasis [34]. These findings show that *CALML3* may be closely related to SKCM metastasis and prognosis, and that it has certain clinical significance for the prediction of SKCM and its prognosis.

Although we identified 11 feature DEGs related to SKCM metastasis, the detailed mechanisms have not yet been explored. For example, a further accurate classification with a large sample size and clinical information needs to identify SKCM metastatic and nonmetastatic patients. In addition, whether the 11 feature DEGs are involved in several molecular pathways, such as hsa04916: Melanogenesis, needs to be investigated. Corresponding experimental research is also needed to verify feature gene functions.

## Conclusion

We identified 876 DEGs (353 downregulated and 523 upregulated) in the TCGA training dataset. In addition, 11 important prognostic-related feature DEGs, such as *ZNF750*, *NLRP6*, *TGM3*, *CALML5*, *CD3G*, and *RTP5*, may play an important role in SKCM metastasis. These DEGs are involved in 9 KEGG signaling pathways, such as the “hsa04916: Melanogenesis” pathway. Of the 11 feature DEGs, *CALML5* is involved in the “hsa04916: Melanogenesis” pathway, 12 targets of which were collected, such as *TGM3*, *PDE1C*, and *CASP14.* This study provides new ideas for exploring the molecular mechanism underlying SKCM metastasis and finding new diagnostic prognostic markers.

## Data Availability

The datasets supporting the conclusions of this article are available in the [GSE65904] and [TCGA] repository, [https://www.ncbi.nlm.nih.gov/geo/query/acc.cgi?acc=GSE65904] and [https://xenabrowser.net/datapages/?cohort=GDC%20TCGA%20Melanoma%20(SKCM)&removeHub=https%3A%2F%2Fxena.treehouse.gi.ucsc.edu%3A443].

## Abbreviations

DEGs: Differentially expressed genes
GO: Gene Ontology
KEGG: Kyoto Encyclopedia of Genes and Genomes
PPI: Protein–protein interaction
SKCM: Skin cutaneous melanoma
TCGA: The Cancer Genome Atlas
